# Supplementary material for: spatialMET: an open and scalable framework for spatial metabolomics analysis
Source: bioRxiv. 2026 Sep 2:2026.08.27.747606. Preprint. [Version 1] doi: 10.64898/2026.08.27.747606 (PMC13684634; doi:10.64898/2026.08.27.747606)
Supplement: Supplementary file 1 [file media-1.pdf]

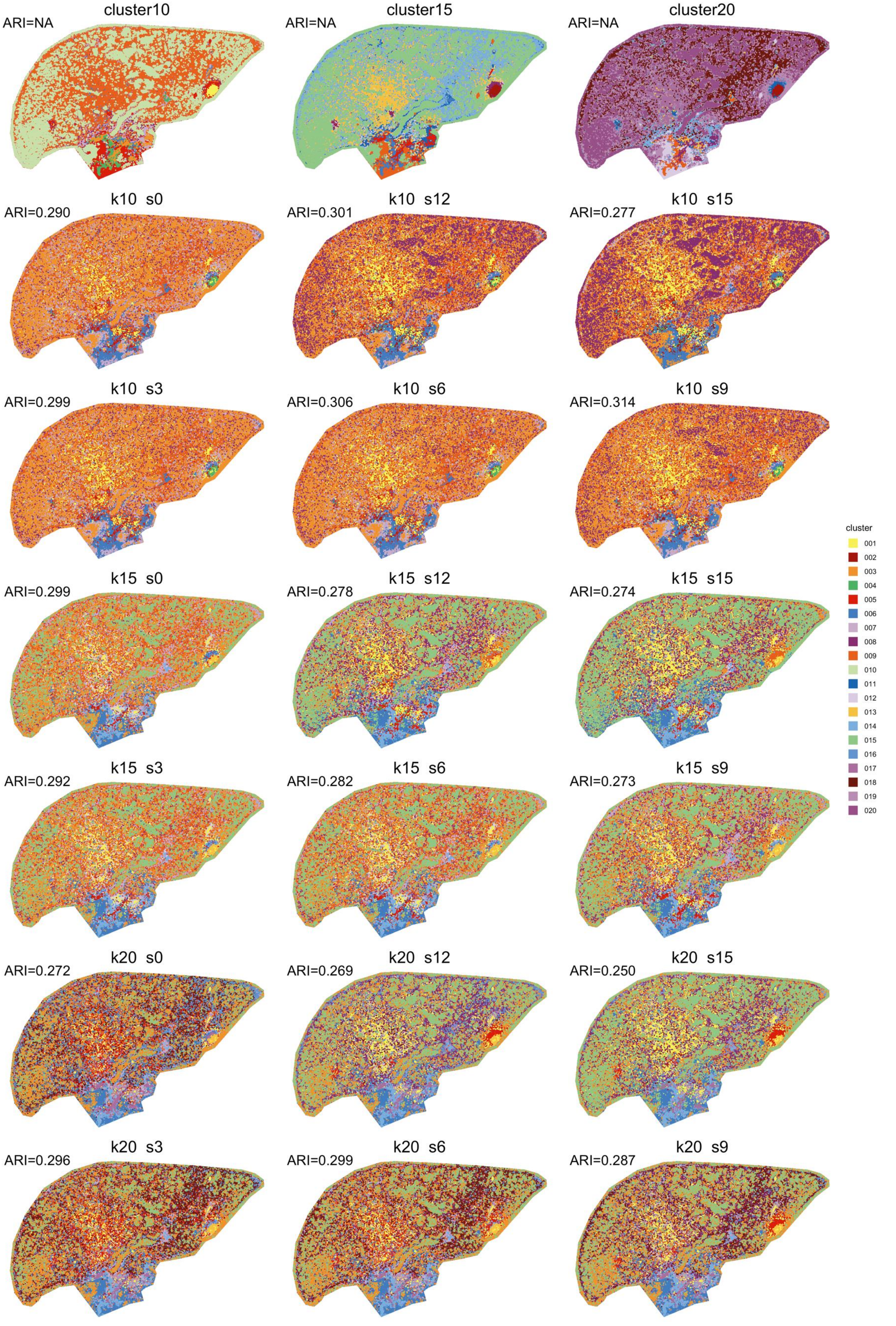

Figure S1. Comparison of pixel-level spatial domain assignments from hcdist and spatial shrunken centroids (SSC). Pixel-level spatial domain assignments are shown for hcdist and SSC across K values of 10, 15, and 20 and SSC sparsity parameters of s = 0, 3, 6, 9, 12, and 15. The adjusted Rand index (ARI) was calculated to measure agreement in spatial domain assignments between the two methods.

.
