# Supplementary material for: spatialMET: an open and scalable framework for spatial metabolomics analysis
Source: bioRxiv. 2026 Sep 2:2026.08.27.747606. Preprint. [Version 1] doi: 10.64898/2026.08.27.747606 (PMC13684634; doi:10.64898/2026.08.27.747606)
Supplement: Supplementary file 2 [file media-2.pdf]

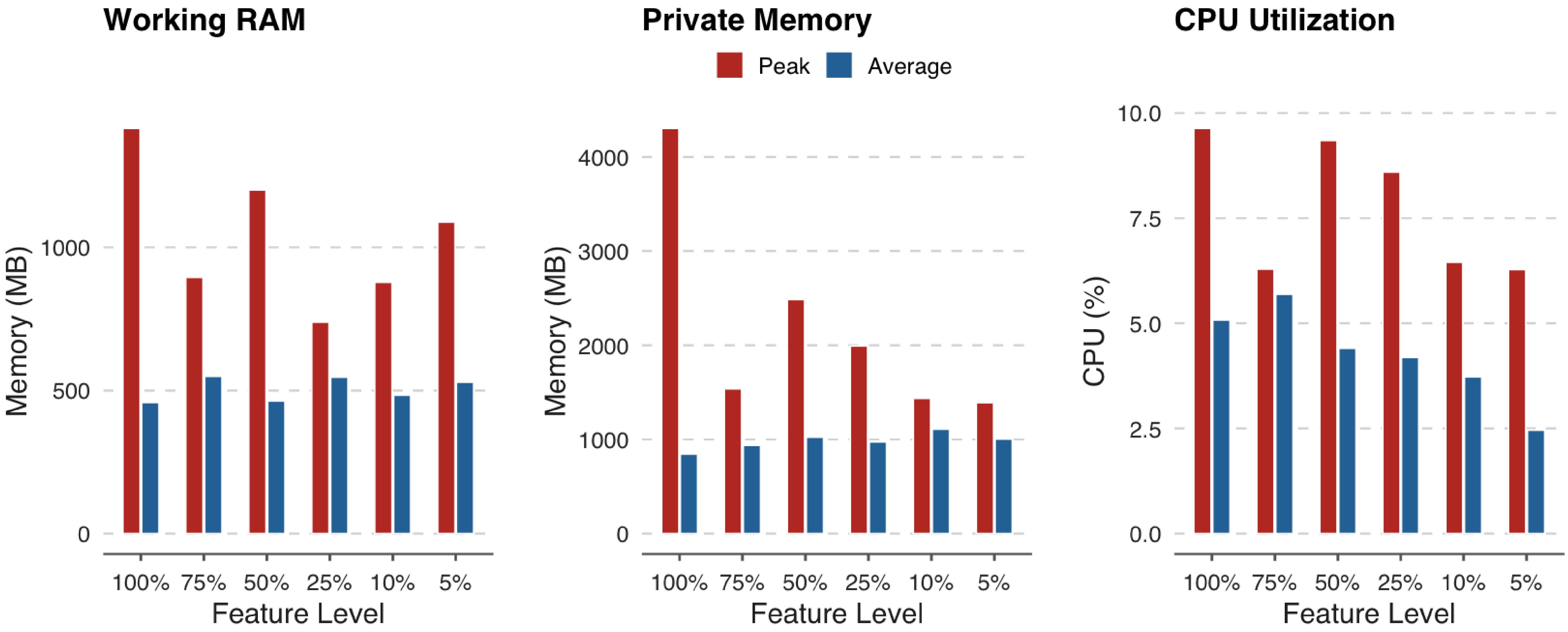

Figure S2. System resource utilization across feature-retention levels. Peak and average working RAM, private memory, and CPU utilization were recorded for six feature-retention levels (100%, 75%, 50%, 25%, 10%, and 5%) using a 5 GB dataset. Peak values are shown in red and average values in blue across all panels.
