# Supplementary material for: spatialMET: an open and scalable framework for spatial metabolomics analysis
Source: bioRxiv. 2026 Sep 2:2026.08.27.747606. Preprint. [Version 1] doi: 10.64898/2026.08.27.747606 (PMC13684634; doi:10.64898/2026.08.27.747606)
Supplement: Supplementary file 4 [file media-4.pdf]

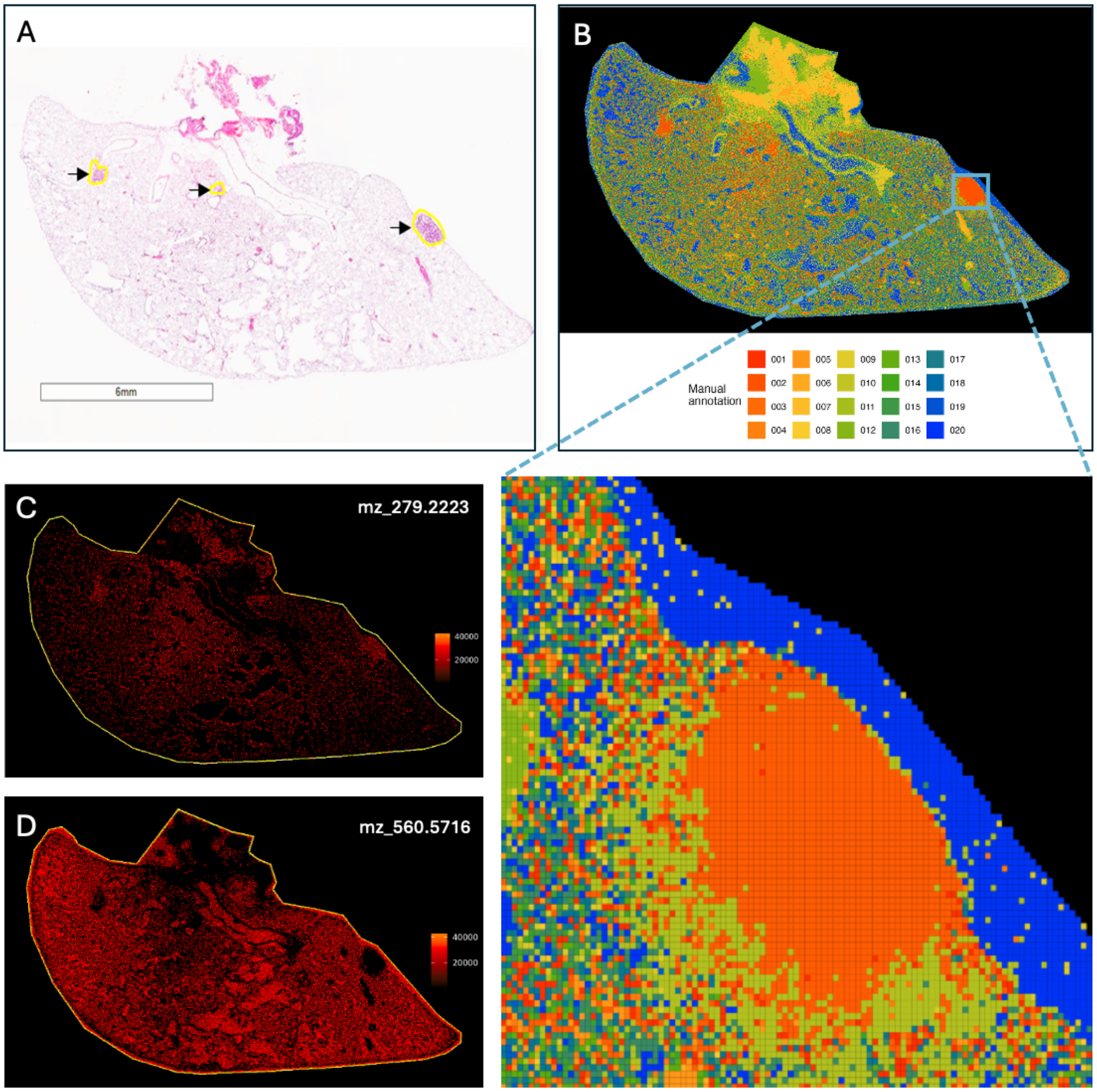

Figure S4. spatialMET identifies tumor-associated spatial domains in non-small cell lung cancer (NSCLC). (A) Hematoxylin and eosin (H&E)-stained tissue section showing histologically defined tumor regions. (B) spatialMET-inferred spatial domains reveal coherent tumor-associated clusters, with a magnified inset highlighting representative domains with distinct spatial boundaries and intradomain heterogeneity. (C) Spatial distribution of m/z feature 279.2223, demonstrating gradual molecular transitions across spatial domains. (D) Spatial distribution of m/z feature 560.5716, illustrating intradomain molecular uniformity within spatialMET-identified regions.
