# Supplementary material for: spatialMET: an open and scalable framework for spatial metabolomics analysis
Source: bioRxiv. 2026 Sep 2:2026.08.27.747606. Preprint. [Version 1] doi: 10.64898/2026.08.27.747606 (PMC13684634; doi:10.64898/2026.08.27.747606)
Supplement: Supplementary file 6 [file media-6.pdf]

Table S1. Comprehensive Comparison of Spatial Metabolomics Software: Benchmarking and Functional Feature Assessment

Category / Metric	spatialMET	Cardinal	Galaxy-MSI	SpaMTP	SpectralAnalysis	Multi-MSIprocessor	SMAnalyst	SMEW
**Data Ingestion Standard**	Native Raw (.imzML, .ibd) & Processed	Raw & Processed (.imzML, .analyze)	Raw & Processed (.raw, .mzML)	imzML, Cardinal Objects	imzML, .raw, .mzML	Raw Vendor Files	Processed Matrix only	Processed Tabular CSV only (Requires external scripts)
**End-to-End Workflow Integration**	Unified Native Pipeline (Raw to Stats)	Scripting required	Fragmented modules	Partial Cardinal dependency	GUI focused	Pre-processing only	Downstream only	Broken Workflow (Requires Stage-1 script pre-processing)
**Pre-processing & QC**	Feature/Pixel QC, Normalisation & Filtering	Baseline & Smoothing	Native Peak Filtering	Supported via Cardinal	Pixel-wise Smoothing	Basic Peak Filtering	Background QC	Native Spatial Denoising & Normalisation
**Dimensionality Reduction**	Integrated PCA & Spatial Coordinates	PCA, PLS	Not Natively Supported	PCA	PCA, NMF	Not Natively Supported	PCA, UMAP, t-SNE	PCA, PLS-DA, NMF, UMAP
**Clustering Capabilities**	High-Performance C-backend (hcdist) & K-means	Spatial Shrunken Centroids	Not Natively Supported	K-means, Hierarchical	Louvain, SLM	K-means	K-means (via Seurat)	K-means, Louvain, BayesSpace
**Differential Abundance**	Native Parametric/Non-Parametric Wilcoxon (Wilcoxon-test, spatial limma, and Hellinger distance)	Native Segmentation	Native t-tests	Native Wilcoxon (FDR)	Native Wilcoxon (FDR)	Not Available	Native Wilcoxon	Native Parametric & Non-Parametric Tests
**Tissue Annotation (ROI)**	Native Interactive Graphical Lasso Tool	Requires R Coding	Requires Manual Mapping	Lacks Native Selection	Lacks Native Selection	Lacks Native Selection	Lacks Native Selection	Native Interactive Selection & H&E Co-registration
**Spatial Gradients**	Native STgradient Module	Lacks Native Module	Lacks Native Module	Lacks Native Module	Lacks Native Module	Lacks Native Module	Lacks Native Module	Native Radial Distance Analysis
**Spatial Autocorrelation**	Native Moran's I & Geary's C	Requires Manual Coding	Lacks Spatial Module	Native SpaGene	Native Moran's I	Non-Spatial FELLA	Lacks Spatial Module	Native semla & MERINGUE
**Spatial Feature Selection**	Supported via Cardinal Integration	Native SSC Selection	Not Supported	Native SpaGene	Not Supported	Not Supported	Not Supported	Native Spatially Variable Peaks
**Biological Concordance / ST Validation**	External/Custom Integration	Custom R Integration	Not Supported	Native Seurat/ST Alignment	Not Supported	Not Supported	Not Supported	Native GENIE3 & MAGPIE ST Alignment
**Metabolite Feature Annotation**	Streamlined Local Reference Mapping (No API Setup)	Requires External Packages	HMDB/METLIN Integration	Internal HMDB/KEGG	Native m/z Matching	Integrated HMDB/METLIN	Not Natively Supported	Requires External Manual Script Setup via REST APIs
**FINAL SCORE**	**10 / 12**	**6 / 12**	**4 / 12**	**8 / 12**	**7 / 12**	**4 / 12**	**4 / 12**	**9/ 12**

Scoring Basis: Each tool is evaluated across the 12 key operational categories. **Point Allocation Criteria (Binary Support Evaluation)**: **1 Point (Native/Integrated Support)**: Awarded when a feature is fully native, out-of-the-box, or integrated seamlessly within the tool's core execution environment (such as GUI-driven interactive lasso selection, native C-compiled backend algorithms, or unified raw-to-stats execution). This corresponds to the green-colored cells in the benchmark matrix. **0 Points (Non-Native/Code-Dependent/Absent)**: Assigned when a feature is either entirely missing, marked as "Lacks native modules", or when support requires external user intervention. This includes workflows described as "Requires manual R coding", "Requires manual coordinate mapping", "Requires external packages", "Requires Stage-1 script pre-processing", or "Requires External Manual Script Setup via REST APIs". This stringent penalty reflects the user-accessibility barrier for non-programmers.
